# Comparing the quantum witness, the entropic Leggett–Garg inequality and the NCGD

**DOI:** 10.1038/s41598-024-60742-y

**Published:** 2024-05-02

**Authors:** Xiangguan Tan, Yuxia Zhang, Tianhui Qiu

**Affiliations:** 1 College of Electronic and Information Engineering, Shandong University of Science and Technology, Qingdao, 266590 China; 2 School of Science, Qingdao University of Technology, Qingdao, 266520 China

**Keywords:** Physics, Quantum physics

## Abstract

In this paper, we investigate the violation of the quantum witness, the entropic Leggett–Garg inequality (LGI) and the no-coherence-generating-and-detecting (NCGD) dynamics, under projective and coarsening measurements. We consider a qubit in the three scenarios: coherent dynamics, in the presence of dissipation, and in the presence of dephasing. For the pure qubit, we find that in the case of the projective measurement, the non-violation conditions of the quantum witness and the NCGD are the same; while the non-violation conditions of the entropic LGI and the quantum witness do not contain each other, i.e., a suitable conjunction of the quantum witness and the entropic LGI may be better for testing macrorealism. Also, for the pure qubit with coarsening measurement similar results can be obtained. For the dissipative qubit with projective measurement, the quantum witness and the NCGD can be both violated for a wider parameter regime than the entropic LGI. For the dissipative qubit with coarsening measurement, the violation of the NCGD is the most robust compared to the quantum witness and the entropic LGI. For the dephasing qubit with projective and coarsening measurements, the relationship among the quantum witness, the entropic LGI and the NCGD is similar to that of the pure qubit. In addition, we find that for pure, dissipative and dephasing qubits, the robustness of the coarsening measurement in final resolution is more vulnerable than that of the coarsening measurement in reference for the entropic LGI.

## Introduction

Quantum physics conceptually and mathematically, is incompatible with a view of the classical world. And the question of what genuinely distinguishes quantum from classical physics is as old as quantum theory itself^1,2^. In other words, how macroscopic classical world emerges from the framework of quantum mechanics (QM) has always been a foundational question, which has been attracting increasing attention. In 1985, Leggett and Garg introduced the concept of macroscopic realism (macrorealism)^3^. Based on the assumption of macrorealism and analogies to Bell’s theorem^4^, Leggett and Garg proposed an inequality, which is now known as the Leggett–Garg inequality (LGI)^3,5,6^. This inequality is proposed to test macrorealism, and probes the correlations of a single system measured at different times. The LGI cannot provide the necessary and sufficient conditions for macrorealism, and is a necessary condition for macrorealism.

For studies along these lines, recently, a quantum witness^7–9^ for probing the non-classical behavior has been proposed. It is based on the classical assumption: the idea that a measurement does not change the outcome statistics of a later measurement^8,9^, which is also known as the non-disturbing-measurement condition^10,11^. In other words, the quantum witness ensures that the outcome statistics of a later measurement does not depend on whether any prior measurement has been performed. Except for the LGI and the quantum witness, there have been other standard tools for testing macrorealism, such as the entropic Leggett–Garg inequality (LGI)^12,13^, coherence-generating-and-detecting (CGD)^14^ dynamics and so on. The entropic LGI recently has been introduced as a criterion to test the incompatibility between the classical world view of macrorealism and QM. It places constraints on the statistical outcomes of temporal correlations of observables. If the entropic LGI is violated, macrorealism is violated. In general, the entropic LGI provides a necessary but not sufficient criterion for local realism and noncontextuality^15,16^. Compared with the quantum witness, the entropic LGI test involves entropies which are functions of correlation probabilities (i.e., it tests macrorealism by using quantities related to probability), and the quantum witness tests macrorealism by directly using probability. In fact, for testing macrorealism, both the entropic LGI and the quantum witness are related to probability. We want to see which one has better robustness when testing macrorealism using quantities related to probability and directly testing macrorealism with probability. In addition, in comparison to a correlation test which involves the probabilities directly, the entropic LGI testing seems to be a less subtle condition. However, in this paper, we find that for the pure and dephasing qubits, in the case of the projective measurement and coarsening measurement, the violation of the quantum witness is not more robust than that of the entropic LGI (see Table 1 and Fig. 2). The other notion of CGD was proposed by Smirne et al.^14^. They presented the property of quantum coherence directly related to the non-classicality possibly emerging from repeated measurements of a quantum observable. Roughly speaking, they characterized the evolutions which not only generate coherences, but can also turn such coherences into the populations measured at a later time. That is to say, when the CGD is satisfied, the evolution can generate coherence, and can turn such coherence into the populations measured at a later time. If the CGD is not satisfied, the evolution can be described as the NCGD. In fact, it provides a definite criterion to determine when and to what extent quantum coherence is equivalent to non-classicality.

Understanding how macrorealism and classical laws can emerge out of quantum physics, has long been a challenging task, leading to significant research efforts. Among these, quantum decoherence is one of the successful attempts to explain the quantum-to-classical transition^17^. Apart from quantum decoherence, a new idea has emerged in recent years, known as the concept of coarsening measurement^18,19^, to explain the quantum-to-classical transition. A coarsening measurement can be divided into coarsening in measurement reference (coarsening the accuracy of this unitary operation) and coarsening in final measurement resolution. This imprecise measurement is a theoretical approach that is conceptually different from the phenomenon of decoherence^19^. In the course of this research trajectory, numerous inquiries have been conducted, contributing a deeper understanding of coarsening measurement resulting in the emergence of classicality or persistence of quantumness^18–32^. And in Refs^18–20,26–32^, these works have done a detailed study on the effects of the coarsening measurement on the LGI. For example, in Ref.^28^, they investigated the violation of the LGI^3,5,6^, Wigner’s form of LGI^33^ and no-signaling in time condition (NSIT)^8,9,34,35^ for multilevel spin systems under the condition of coarsening measurement. They found that the effect of coarsening of measurement times in reducing the magnitude of quantum violation of macrorealism can be compensated by increasing the dimension of the quantum system. However, we rarely see reports on the effects of coarsening measurement on the quantum witness, the entropic LGI and the NCGD, and about comparing the robustness of these three conditions of macrorealism.

In this paper, we discuss the effects of projective and coarsening measurements on the quantum witness, the entropic LGI and the NCGD for a qubit in three scenarios: with coherent dynamics, dissipative dynamics and dephasing dynamics. And then, we investigate the violation of these three different conditions of macrorealism, to find a stricter criterion for testing macrorealism. The coarsening measurement contains coarsening measurement in reference and final resolution. For the pure qubit with projective measurement, we find that the non-violation conditions of the NCGD are the same as the quantum witness; and a suitable conjunction of the quantum witness and the entropic LGI may be better for testing macrorealism. Also, for the pure qubit with coarsening measurement similar results can be obtained. For the dissipative qubit with projective measurement, the non-violation of the quantum witness and the NCGD implies the non-violation of the entropic LGI. Then, for the dissipative qubit with coarsening measurement, the violation of the NCGD is the most robust and the quantum witness lies between the NCGD and the entropic LGI, and the violation of the entropic LGI is the most vulnerable. For the dephasing qubit with projective and coarsening measurements, the relationship among the quantum witness, the entropic LGI and the NCGD is similar to that of the pure qubit. In addition, we find that for the entropic LGI, the violation of the coarsening measurement reference is more robust than that of the coarsening measurement final resolution, in the case of pure, dissipative and dephasing qubits.

## Coarsening measurement

We firstly briefly review the coarsening measurement^18,19^. The coarsening measurement contains the coarsening measurement in measurement reference and the coarsening measurement in final measurement resolution^18,19^. Consider a qubit observable $\sigma _{z}$, and its projector is described as

1 $$\begin{aligned} {{\Pi }}_{z}^{a_{z}} =\frac{1}{2}(I+a_{z}{\sigma _{z}}), \end{aligned}$$

where $a_{z}=\pm 1$ are the outcomes of observable $\sigma _{z}$. Here, ${{\Pi }}_{z}^{a_{z}=+1}=|0\rangle \langle 0|$, and ${{\Pi }}_{z}^{a_{z}=-1}=|1\rangle \langle 1|$, where $|0\rangle$ and $|1\rangle$ are the eigenvectors of the observable $\sigma _{z}$, respectively. Next, when the final measurement resolution is coarsened, the corresponding fuzzy version of the measurement operators can be described as

2 $$\begin{aligned} {\Pi }_{{ {z}},\delta }^{{ {a_{z}=}}+1}= & {} {\Pi }_{{ {z}},\delta }^{+} =(1-\delta )|0\rangle \langle 0|+\delta |1\rangle \langle 1|,\nonumber \\ {\Pi }_{{ {z}},\delta }^{{ {a_{z}=}}-1}= & {} {\Pi }_{{ {z}},\delta }^{-} =(1-\delta )|1\rangle \langle 1|+\delta |0\rangle \langle 0|. \end{aligned}$$

Here, $\delta$ is the coarsening degree (or, degree of fuzziness) of the final measurement resolution ($0<\delta <0.5$).

Then, we show another version of the coarsening measurement, i.e., the coarsening measurement in reference. The corresponding measurement operator can be denoted as

3 $$\begin{aligned} {{\Pi }}_{z,{{\Delta }}}^{{ {a}}_z}{ = \iint \limits ^{+\infty }_{-\infty }{d\theta d\varphi {\lambda _{{\Delta }} }(\theta - {\theta _0})} }\lambda _{{{\Delta }}}(\varphi -\varphi _{0}) U {(\theta , \varphi )^\dagger }{{\Pi }}_{z,\delta }^{{ {a}}_z}U(\theta , \varphi ). \end{aligned}$$

Here, $U(\theta , \varphi )$ is a unitary operator and implies a rotation of measurement axes about *y* axis and *z* axis:

4 $$\begin{aligned} U(\theta , \varphi )\left| 0 \right\rangle= & {} \left| {{o_n}} \right\rangle = \cos \frac{\theta }{2}\left| 0 \right\rangle + {e^{i\varphi }}\sin \frac{\theta }{2}\left| 1 \right\rangle , \nonumber \\ U(\theta , \varphi )\left| 1 \right\rangle= & {} \left| {{o_{ - n}}} \right\rangle = {e^{ - i\varphi }}\sin \frac{\theta }{2}\left| 0 \right\rangle - \cos \frac{\theta }{2}\left| 1 \right\rangle , \end{aligned}$$

where $\left| {{o_{\pm n}}} \right\rangle$ are the eigenvectors of $\sigma _{n}={{\varvec{n}}}\cdot \varvec{\sigma }$. And ${{\varvec{n}}}=\sin \theta \cos \varphi \,{{\varvec{i}}}+\sin \theta \sin \varphi \, {{\varvec{j}}}+\cos \theta \, {{\varvec{k}}}$ is a unit vector in the Bloch sphere, and $\varvec{\sigma }=\sigma _{x} \,{{\varvec{i}}}+\sigma _{y} \, {{\varvec{j}}}+\sigma _{z}\, {{\varvec{k}}}$ ($\sigma _{x}$, $\sigma _{y}$ and $\sigma _{z}$ are Pauli operators). And ${\lambda _{{\Delta }} }(\theta - {\theta _0})$ and ${\lambda _{{\Delta }} }(\varphi -\varphi _{0})$ in Eq. (3) are the normalized Gaussian kernels with standard deviation ${\Delta }$, which are centered around $\theta _{0}$ and $\varphi _{0}$, respectively. Similarly, ${\Delta }$ is the coarsening degree (or, degree of fuzziness) of the measurement reference ($0<{{\Delta }}<1$). These normalized Gaussian kernels (in Eq. (3)) satisfy ${\lambda _{{\Delta }} }(\theta - {\theta _0}) = \frac{1}{{\sqrt{2\pi } {{\Delta }} }}$exp$[{{ - \frac{{{{(\theta - {\theta _0})}^2}}}{{2{{{\Delta }} ^2}}}}}]$ and ${\lambda _{{\Delta }} }(\varphi -\varphi _{0}) = \frac{1}{{\sqrt{2\pi } {{\Delta }} }}$exp$[{{ - \frac{{{{(\varphi -\varphi _{0})}^2}}}{{2{{{\Delta }} ^2}}}}}]$, respectively.

## A pure qubit

### Quantum witness

Firstly, we briefly recapitulate the quantum witness^7^, which has been introduced in two slightly different ways in Refs.^8,9^. Following Ref.^7^, we consider a two-level system ($d=2$ in Ref.^7^), and suppose that Hamiltonian satisfies $H=\frac{1}{2}\omega \sigma _{x}$, where $\omega$ is the energy gap of the qubit. And the system unitary evolution operator between $t_{i}$ and $t_{j}$ can be given by $U(t_j, t_i)=e^{-i H \tau }=e^{-\frac{i}{2}\omega \sigma _{x}( t_{j}-t_{i})}$, with $\tau = t_{j}-t_{i}$. For simplicity, we suppose $\tau \in [0, \dfrac{\pi }{\omega }]$ in the following. Then, at different times $t_i$ and $t_j$ ($i, j=0, 1, 2$ and $t_i< t_j$), we perform the sequential measurements of ${{\Pi }}^{n}(t_i)$ and ${{\Pi }}^{m}(t_j)$, respectively. Here, $n, m=\pm 1$ are outcomes of the observable. Then, the quantum witness can be expressed as^8^

5 $$\begin{aligned} W_{q}=\vert P({{\Pi }}^{m}(t_j)) - \sum _{{n}} P({{\Pi }}^{n}(t_i),{{\Pi }}^{m}(t_j))\vert , \end{aligned}$$

where $P({{\Pi }}^{m}(t_j))$ is the probability of obtaining outcome *m* by measuring at $t_{j}$, and $P({{\Pi }}^{n}(t_i),{{\Pi }}^{m}(t_j))$ is the probability of obtaining outcomes *n* and *m* for measurements at two instants $t_{i}$ and $t_{j}$, respectively. And the probability $P({{\Pi }}^{n}(t_i),{{\Pi }}^{m}(t_j))$ can be expressed as

6 $$\begin{aligned} P(\Pi ^{n}(t_i),\Pi ^{m}(t_j))={\textrm{Tr}}[\Pi ^{m}(t_j)U(t_{j},t_{i})\Pi ^{n}(t_i)U(t_{i},{t_{0}}) \rho (0) { {U}}(t_{i},t_{0})^{\dag }\Pi ^{n}(t_i)^{\dag } { {U}(t_{j},t_{i})^{\dag }} \Pi ^{m}(t_j)^{\dag }], \end{aligned}$$

where $\rho (0)$ is the initial state of the system. The non-classicality of the initial state is revealed, when $W_q>0$. And when $W_q=0$, the quantum witness is satisfied. It is worth noting that when one of the quantum witnesses is violated, macrorealism is violated.

Next, we investigate the quantum witness under the projective measurement (in Eq. (1)) in Schr$\ddot{\mathrm{o}}$dinger’s picture. The initial state of the system at $t_0 = 0$ can be written as $\rho (0)=\frac{1-\alpha }{2}|0\rangle \langle 0|+\frac{1+\alpha }{2}|1\rangle \langle 1|$, with $0\le \alpha \le 1$. From Eqs. (1), (5) and (6), we obtain all the quantum witnesses under the projective measurement. For the sake of simplicity, we take the quantum witness: $W_{q}=\mid P({{\Pi }}^{+}(t_2)) - \sum _{{\pm }} P({{\Pi }}^{\pm }(t_1),{{\Pi }}^{+}(t_2))\mid$ as an example to illustrate, in this paper. Then, it can be expressed as

7 $$\begin{aligned} W_{q}=\mid P({{\Pi }}^{+}(t_2)) - \sum _{{\pm }} P({{\Pi }}^{\pm }(t_1),{{\Pi }}^{+}(t_2))\mid =\frac{1}{2} \alpha \sin ^2 \omega \tau . \end{aligned}$$

From the above expression, we find that when one of the conditions is satisfied: (1) $\tau =\frac{\pi }{\omega }$; (2) $\alpha =0$, $W_{q}$ in Eq. (7) will equal to zero, i.e., the quantum witness will be satisfied. And other quantum witnesses are similar to Eq. (7). Therefore, all the quantum witnesses will be satisfied, when one of the conditions is satisfied: (1) $\tau =\frac{\pi }{\omega }$; (2) $\alpha =0$. These non-violation conditions are listed in Table 1. Next, we investigate the quantum witness under the coarsening measurement in reference (${{\Delta }}\ne 0, \delta =0$). Similarly, we also take $W_{q,{{\Delta }}}=\mid P_{{\Delta }}({{\Pi }}^{+}(t_2)) - \sum _{{\pm }} P_{{{\Delta }}}({{\Pi }}^{\pm }(t_1),{{\Pi }}^{+}(t_2))\mid$ as an example. From Eqs. (3), (5) and (6), $W_{q,{{\Delta }}}=\mid P_{{\Delta }}({{\Pi }}^{+}(t_2)) - \sum _{{\pm }} P_{{{\Delta }}}({{\Pi }}^{\pm }(t_1),{{\Pi }}^{+}(t_2))\mid$ can be obtained as

8 $$\begin{aligned} W_{q, {{\Delta }}}=\mid P_{{\Delta }}({{\Pi }}^{+}(t_2)) - \sum _{{\pm }} P_{{\Delta }}({{\Pi }}^{\pm }(t_1),{{\Pi }}^{+}(t_2))\mid =-\frac{1}{2} \alpha e^{-\frac{{{\Delta }} ^2}{2}} \left( \sqrt{1-e^{-{{\Delta }} ^2}}-1\right) \sin ^2 \omega \tau , \end{aligned}$$

and other quantum witnesses are similar to it. It can be found from Eq. (8) that if one of the conditions is satisfied: (1)$\tau =\frac{\pi }{\omega }$; (2) $\alpha =0$, the quantum witness will be satisfied. Therefore, when one of the conditions is satisfied: (1) $\tau =\frac{\pi }{\omega }$; (2) $\alpha =0$, all the quantum witnesses will not be violated. Similarly, these non-violation conditions are summarized in Table 1. Now, we discuss the quantum witness under the coarsening measurement in final resolution (Eq. (2)). From Eqs. (2), (5) and (6), the quantum witness: $W_{q,\delta }=\mid P_\delta ({{\Pi }}^{+}(t_2)) - \sum _{{\pm }} P_\delta ({{\Pi }}^{\pm }(t_1),{{\Pi }}^{+}(t_2))\mid$ can be denoted as

9 $$\begin{aligned} W_{q,\delta }=\mid P_\delta ({{\Pi }}^{+}(t_2)) - \sum _{{\pm }} P_\delta ({{\Pi }}^{\pm }(t_1),{{\Pi }}^{+}(t_2))\mid =\frac{1}{2} \alpha (2 \delta -1) \left[ 2 \sqrt{-(\delta -1) \delta }-1\right] \sin ^2 \omega \tau . \end{aligned}$$

Others are similar to the above expression. From Eq. (9), it can be found that if one of the conditions is satisfied: (1) $\tau =\frac{\pi }{\omega }$; (2) $\alpha =0$, the quantum witness will not be violated. Thus, all the quantum witnesses will be satisfied, when one of the conditions is satisfied: (1) $\tau =\frac{\pi }{\omega }$; (2) $\alpha =0$, which are listed in Table 1.

**Table 1:** Non-violation conditions of the quantum witness, the entropic LGI and the NCGD for the coherent dynamics, dynamics with dissipation and with dephasing, in the case of projective and coarsening measurements ($0<\Delta <1$ and $0<\delta <0.5$), respectively.

Measurement operator	Quantum witness	Entropic LGI	NCGD
Coherent dynamics			
Projective measurement	(1) $\tau =\frac{\pi }{\omega }$;	(1) $\tau =\frac{\pi }{\omega }$;	(1) $\tau =\frac{\pi }{\omega }$;
		(2) $\alpha =0$ and $0.2062\frac{\pi }{\omega }\le \tau \le 0.7938\frac{\pi }{\omega }$;	
	(2) $\alpha =0$;	(3) $\alpha =0.5$ and $0.4465\frac{\pi }{\omega }\le \tau \le 0.5535\frac{\pi }{\omega }$;	(2) $\alpha =0$;
Coarsening measurement In reference	(1) $\tau =\frac{\pi }{\omega }$;	(1) $\tau =\frac{\pi }{\omega }$;	(1) $\tau =\frac{\pi }{\omega }$;
		(2) $\alpha =0$, $\tau =0.1\frac{\pi }{\omega }$ and $0.19235\le \Delta <1$;	
	(2) $\alpha =0$;	(3) $\alpha =0.5$, $\tau =0.1\frac{\pi }{\omega }$ and $0.19359\le \Delta <1$;	(2) $\alpha =0$;
Coarsening measurement In final resolution	(1) $\tau =\frac{\pi }{\omega }$;	(1) $\tau =\frac{\pi }{\omega }$;	(1) $\tau =\frac{\pi }{\omega }$;
		(2) $\alpha =0$, $\tau =0.1\frac{\pi }{\omega }$ and $0.00917\le \delta <0.5$;	
	(2) $\alpha =0$;	(3) $\alpha =0.5$, $\tau =0.1\frac{\pi }{\omega }$ and $0.0093\le \delta <0.5$;	(2) $\alpha =0$;
Dynamics with dissipation $(\tau =\frac{\pi }{2\omega })$			
Projective measurement	$\alpha =e^{\frac{2 \pi \gamma }{\omega }}-1$	(1) $\alpha =e^{\frac{2 \pi \gamma }{\omega }}-1$ ($\gamma \in (0, \frac{\log 2}{2 \pi }\omega ]$);	$\alpha =e^{\frac{2 \pi \gamma }{\omega }}-1$
		(2) $\alpha =0$;	
	($\gamma \in (0, \frac{\log 2}{2 \pi }\omega ]$);	(3) $\alpha =0.5$ and $0.00684\omega \le \gamma \le 0.50432\omega$;	($\gamma \in (0, \frac{\log 2}{2 \pi }\omega ]$);
Coarsening measurement in reference	$\alpha =-\frac{\left( e^{\frac{\pi \gamma }{\omega }}-1\right) \left( \sqrt{1-e^{-\Delta ^2}} \left( -e^{\frac{\pi \gamma }{2 \omega }}\right) +e^{\frac{\pi \gamma }{\omega }}+1\right) }{\sqrt{1-e^{-\Delta ^2}} e^{\frac{\pi \gamma }{2 \omega }}-1}$	(1) $\alpha =-\frac{\left( e^{\frac{\pi \gamma }{\omega }}-1\right) \left( \sqrt{1-e^{-\Delta ^2}} \left( -e^{\frac{\pi \gamma }{2 \omega }}\right) +e^{\frac{\pi \gamma }{\omega }}+1\right) }{\sqrt{1-e^{-\Delta ^2}} e^{\frac{\pi \gamma }{2 \omega }}-1}$ ($\alpha \in [0, 1]$);	No
		(2) $\alpha =0$;	
	($\alpha \in [0, 1]$);	(3) $\alpha =0.5$, $\gamma =0.6\omega$ and $0.20522\le \Delta <1$;	
Coarsening measurement in final resolution		(1) $\alpha =-\frac{\left( e^{\frac{\pi \gamma }{\omega }}-1\right) \left( -2 \sqrt{-(\delta -1) \delta } e^{\frac{\pi \gamma }{2 \omega }}+e^{\frac{\pi \gamma }{\omega }}+1\right) }{2 \sqrt{-(\delta -1) \delta } e^{\frac{\pi \gamma }{2 \omega }}-1}$ ($\alpha \in [0, 1]$);	No
	$\alpha =-\frac{\left( e^{\frac{\pi \gamma }{\omega }}-1\right) \left( -2 \sqrt{-(\delta -1) \delta } e^{\frac{\pi \gamma }{2 \omega }}+e^{\frac{\pi \gamma }{\omega }}+1\right) }{2 \sqrt{-(\delta -1) \delta } e^{\frac{\pi \gamma }{2 \omega }}-1}$	(2) $\alpha =0$;	
	($\alpha \in [0, 1]$);	(3) $\alpha =0.5$, $\gamma =0.6\omega$ and $0.01042\le \delta <0.5$;	
Dynamics with dephasing			
	(1) $\tau =\frac{\pi }{\omega }$;	(1) $\tau =\frac{\pi }{\omega }$;	(1) $\tau =\frac{\pi }{\omega }$;
Projective measurement		(2) $\alpha =0$ and $0.2062\frac{\pi }{\omega }\le \tau \le 0.7938\frac{\pi }{\omega }$;	
	(2) $\alpha =0$;	(3) $\alpha =0.5$ and $0.4465\frac{\pi }{\omega }\le \tau \le 0.5535\frac{\pi }{\omega }$;	(2) $\alpha =0$;
Coarsening measurement in reference	(1) $\tau =\frac{\pi }{\omega }$;	(1) $\tau =\frac{\pi }{\omega }$;	(1) $\tau =\frac{\pi }{\omega }$;
		(2) $\alpha =0$, $\tau =0.1\frac{\pi }{\omega }$ and $0.19235\le \Delta <1$;	
	(2) $\alpha =0$;	(3) $\alpha =0.5$, $\tau =0.1\frac{\pi }{\omega }$, $\gamma =0.6\omega$ and $0.19405\le \Delta <1$;	(2) $\alpha =0$;
Coarsening measurement in final resolution	(1) $\tau =\frac{\pi }{\omega }$;	(1) $\tau =\frac{\pi }{\omega }$;	(1) $\tau =\frac{\pi }{\omega }$;
		(2) $\alpha =0$, $\tau =0.1\frac{\pi }{\omega }$ and $0.00917\le \delta <0.5$;	
	(2) $\alpha =0$;	(3) $\alpha =0.5$, $\tau =0.1\frac{\pi }{\omega }$, $\gamma =0.6\omega$ and $0.00933\le \delta <0.5$;	(2) $\alpha =0$;

### Entropic LGI

Next, we introduce the entropic LGI as discussed in references^12,13^. In classical information theory, the properties of Shannon entropy, including the chain rule, can be described as follows: $H({\Pi }^{n}(t_i), {\Pi }^{m}(t_j))=H({\Pi }^{n}(t_i)\vert {\Pi }^{m}(t_j))+H( {\Pi }^{m}(t_j))=H({\Pi }^{m}(t_j)\vert {\Pi }^{n}(t_i))+H({\Pi }^{n}(t_i))$, and $H({\Pi }^{n}(t_i), {\Pi }^{m}(t_j))\le H({\Pi }^{n}(t_i))+H({\Pi }^{m}(t_j))$. From the properties of Shannon entropy, we can derive the following inequalities: $H({\Pi }^{n}(t_i)\vert {\Pi }^{m}(t_j))\le H({\Pi }^{n}(t_i))$ and $H({\Pi }^{m}(t_j)\vert {\Pi }^{n}(t_i))\le H({\Pi }^{m}(t_j))$. These inequalities imply that if conditions are imposed, the information of random variables will be reduced. Using the chain rule, the joint Shannon entropy for three observables $\Pi ^{n}(t_0)$, $\Pi ^{m}(t_1)$, and $\Pi ^{l}(t_2)$ at $t_0$, $t_1$, and $t_2$, respectively, can be written as follows: $H(\Pi ^{n}(t_0), \Pi ^{m}(t_1), \Pi ^{l}(t_2))=H(\Pi ^{l}(t_2)\vert \Pi ^{m}(t_1), \Pi ^{n}(t_0))+H(\Pi ^{m}(t_1)\vert \Pi ^{n}(t_0))+H(\Pi ^{n}(t_0))$, with $l=\pm 1$ being measurement outcomes of observable at $t_2$. From these properties of Shannon entropy, the entropic LGI can be obtained as^13^

10 $$\begin{aligned} H_{1}=H(\Pi ^{n}(t_0), \Pi ^{l}(t_2))-H(\Pi ^{m}(t_1),\Pi ^{l}(t_2))-H(\Pi ^{n}(t_0), \Pi ^{m}(t_1))+H(\Pi ^{m}(t_1)), \end{aligned}$$

where $H({\Pi }^{n}(t_i))=-\sum _{n=\pm 1}P({\Pi }^{n}(t_i))\ln P({\Pi }^{n}(t_i))$, and $H({\Pi }^{n}(t_i), {\Pi }^{m}(t_j))=-\sum _{n,m=\pm 1}P({\Pi }^{n}(t_i),{\Pi }^{m}(t_j))\ln P({\Pi }^{n}(t_i),{\Pi }^{m}(t_j) )$ ($i, j=0, 1, 2$ and $i< j$). Here, *n*, *m* and *l* are the measurement outcomes. The other two entropic LGIs, i.e., $H_{2}$ and $H_{3}$, can be attained by a similar method above. Macrorealism can be satisfied when $H_{1}$, $H_{2}$ and $H_{3}$ are all less than or equal to 0 (i.e., when $H_{1}\le 0$, $H_{2}\le 0$ and $H_{3}\le 0$, macrorealism is satisfied). In other words, when one of the entropic LGIs is violated, macrorealism is violated.

Now, we investigate the entropic LGI under the projective measurement. From Eqs. (1), (6) and (10), we obtain entropic LGIs $H_{1}$, $H_{2}$ and $H_{3}$ as following:

11 $$\begin{aligned} H_{1}=\frac{1}{2} \left[ \log \left( \frac{1}{16} \tan ^2\tau \omega \right) -\cos 2\tau \omega \log \left( \cot ^2\tau \omega \right) +2 \cos \tau \omega \left( \tanh ^{-1}(\cos \tau \omega )+\log (\cot \left( \frac{\tau \omega }{2}\right) )\right) \right] , \end{aligned}$$

12 $$\begin{aligned}&H_{2}=\frac{1}{2} \left[ 2 \alpha \tanh ^{-1}\alpha +\log \left( \frac{\left( \alpha ^2-1\right) \sin ^2 2 \tau \omega }{4 \alpha ^2 \cos ^2\tau \omega -4}\right) +2 \cos \tau \omega \left( -\alpha \tanh ^{-1}(\alpha \cos \tau \omega )+\log (\tan \left( \frac{\tau \omega }{2}\right) )+\tanh ^{-1}(\cos \tau \omega )\right) +\cos 2 \tau \omega \log \left( \cot ^2\tau \omega \right) \right] , \end{aligned}$$

13 $$\begin{aligned}&H_{3}=\frac{1}{2}\left[ \log \left( \frac{2 \sin ^2\tau \omega \cos ^2\tau \omega \left( \alpha ^2 \cos ^2\tau \omega -1\right) }{\alpha ^2+\alpha ^2 \cos 4 \tau \omega -2}\right) +\cos 2 \tau \omega \left( \log \left( \cot ^2\tau \omega \right) -2 \alpha \tanh ^{-1}(\alpha \cos 2 \tau \omega )\right) \right. \nonumber \\&\left. -2 \cos \tau \omega \left( -\alpha \tanh ^{-1}(\alpha \cos \tau \omega )+\log \left( \tan \left( \frac{\tau \omega }{2}\right) \right) +\tanh ^{-1}(\cos \tau \omega )\right) \right] . \end{aligned}$$

From Eqs. (11–13), we find the non-violation conditions for the entropic LGI. Due to the numerous and complex non-violation conditions of the entropic LGI, it is too cumbersome to list every term of non-violation conditions of the entropic LGI. Therefore, we do not list all non-violation conditions, and only a portion of them are listed in Table 1, i.e., (1) $\tau =\frac{\pi }{\omega }$; (2) $\alpha =0$ and $0.2062\frac{\pi }{\omega }\le \tau \le 0.7938\frac{\pi }{\omega }$; (3) $\alpha =0.5$ and $0.4465\frac{\pi }{\omega }\le \tau \le 0.5535\frac{\pi }{\omega }$ (Selecting these conditions as examples is to facilitate comparison with the non-violation conditions of the quantum witness). That is to say, the entropic LGI will be satisfied, when one of the conditions is satisfied: (1) $\tau =\frac{\pi }{\omega }$; (2) $\alpha =0$ and $0.2062\frac{\pi }{\omega }\le \tau \le 0.7938\frac{\pi }{\omega }$; (3) $\alpha =0.5$ and $0.4465\frac{\pi }{\omega }\le \tau \le 0.5535\frac{\pi }{\omega }$.

**Figure 1:**
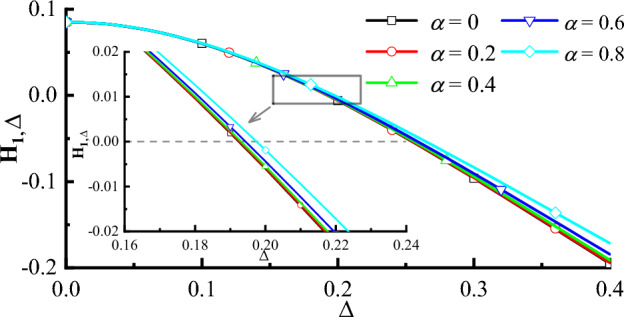
The entropic LG, $H_{1, \Delta }$, as a function of $\Delta$ in coarsening measurement reference under different values of $\alpha =0, 0.2,$ 0.4, 0.6, 0.8 for the pure qubit with $\tau =0.1\frac{\pi }{\omega }$ (black solid line for $\alpha =0$, red solid line for $\alpha =0.2$, green solid line for $\alpha =0.4$, blue solid line for $\alpha =0.6$ and baby blue solid line for $\alpha =0.8$), respectively. For the sake of clarity, we have included a small graph in the figure to illustrate the different values of $\alpha$. The small graph displays five different lines: a black solid line representing $\alpha =0$, a red solid line representing $\alpha =0.2$, a green solid line representing $\alpha =0.4$, a blue solid line representing $\alpha =0.6$ and a baby blue solid line representing $\alpha =0.8$. And the black dashed line in the small graph indicates the classical limit of the entropic LGI, 0.

Next, if the measurement is the coarsening measurement reference ($\Delta \ne 0$ and $\delta = 0$), we obtain the entropic LGI and non-violation conditions of it. Similarly, for simplicity, we only list partial non-violation conditions in Table 1. Then, we define the value of making all the entropic LGIs equal to zero (i.e., $H_{1, \Delta }=H_{2, \Delta }=H_{3, \Delta }=0$) as a critical value of the entropic LGI, which is denoted as $\Delta _{critical}$. And when $\Delta <\Delta _{critical}$, the entropic LGI will be violated. Now, we suppose $\tau =0.1\frac{\pi }{\omega }$ and $\alpha =0, 0.1, 0.2, 0.3, 0.4, 0.5, 0.6, 0.7, 0.8, 0.9, 1$ to discuss the critical values of the entropic LGI. For different values of $\alpha$ ($\alpha =0, 0.1, 0.2, 0.3, 0.4, 0.5, 0.6, 0.7, 0.8, 0.9, 1$) and $\tau =0.1\frac{\pi }{\omega }$, the critical values of the entropic LGI in the coarsening measurement reference, are obtained and then listed in Table 2. From Table 2, we find that as the value of $\alpha$ increases, the critical value of the entropic LGI increases, when the measurement reference is coarsened. Then, we plot Fig. 1 to show that the entropic LG, $H_{1, \Delta }$, as a function of $\Delta$ in the coarsening measurement reference with $\alpha =0, 0.2, 0.4, 0.6, 0.8$ for $\tau =0.1\frac{\pi }{\omega }$. It can be found from Fig. 1 that as the degree of coarsening in measurement reference $\Delta$ increases, the value of the entropic LG function decreases.

Next, when the final measurement resolution is coarsened, we obtain the entropic LGI, and then list partial non-violation conditions of it in Table 1. Then, we define the value of making all the entropic LGIs equal to zero (i.e., $H_{1, \delta }=H_{2, \delta }=H_{3, \delta }=0$) as the critical value of the entropic LGI, i.e., $\delta _{critical}$. And when $\delta <\delta _{critical}$, the entropic LGI will not be satisfied. Similarly, we also suppose $\tau =0.1\frac{\pi }{\omega }$ and $\alpha =0, 0.1, 0.2, 0.3, 0.4, 0.5, 0.6, 0.7, 0.8, 0.9, 1$ to study the critical values of the entropic LGI. We list the critical values of the entropic LGI under the coarsening final measurement resolution, for $\alpha =0, 0.1, 0.2, 0.3, 0.4, 0.5, 0.6, 0.7, 0.8, 0.9, 1$ and $\tau =0.1\frac{\pi }{\omega }$, in Table 2. It can be seen from Table 2 that for the coarsening final measurement resolution, the critical value of the entropic LGI increases with the value of $\alpha$ increasing, which is similar to that of the coarsening of measurement reference. Moreover, for different $\alpha$, we find that similar to the entropic LGI in coarsening of measurement reference, the entropic LG function $H_{1, \delta }$ also decreases with the degree of coarsening in final resolution $\delta$ increasing. In addition, comparing the critical values of the entropic LGI for the coarsening measurement reference and the coarsening final measurement resolution, we found from Table 2 that the critical values of the entropic LGI with $\alpha =0$ for the coarsening measurement reference and the coarsening final measurement resolution are $\Delta _{critical}=0.19235$ and $\delta _{critical}=0.009164$, respectively. That is to say, the entropic LGI for the coarsening measurement in reference can be violated when $\Delta <0.19235$, and the entropic LGI for the coarsening measurement in final resolution can be violated when $\delta <0.009164$. Therefore, the entropic LGI for the coarsening measurement in reference can be violated for a wider parameter than that of the coarsening measurement in final resolution. Other situations (for different $\alpha$ in Table 2) are similar to the above situation. In a word, the violation of the coarsening measurement reference is more robust than that of the coarsening final measurement resolution.

**Table 2:** The critical values of the entropic LGI in the case of $\tau =0.1\frac{\pi }{\omega }$ with $\alpha =0, 0.1, 0.2, 0.3, 0.4, 0.5, 0.6, 0.7, 0.8,$ 0.9, 1, for the pure qubit under the coarsening measurement reference and the coarsening final measurement resolution, respectively.

$\alpha$	$\Delta _{critical}$	$\delta _{critical}$
0	0.19235	0.009164
0.1	0.19239	0.009168
0.2	0.19252	0.009181
0.3	0.19274	0.009202
0.4	0.19308	0.009235
0.5	0.19359	0.009283
0.6	0.19434	0.009355
0.7	0.1955	0.009466
0.8	0.19744	0.009652
0.9	0.20126	0.010025
1	0.21678	0.011611

### NCGD

Next, we briefly introduce the NCGD^14^, which can be described as $\mathrm{\Lambda } \cdot U (t_j, t_i)\cdot \mathrm{\Lambda }\cdot U (t_i, t_0)\cdot \mathrm{\Lambda } \cdot \rho (0)=\mathrm{\Lambda } \cdot U (t_j, t_0)\cdot \mathrm{\Lambda } \cdot \rho (0)$. For simplicity, we define the following quantity:

14 $$\begin{aligned} N=\mathrm{\Lambda } \cdot U (t_j, t_i)\cdot \mathrm{\Lambda }\cdot U (t_i, t_0)\cdot \mathrm{\Lambda } \cdot \rho (0)-\mathrm{\Lambda } \cdot U (t_j, t_0)\cdot \mathrm{\Lambda } \cdot \rho (0), \end{aligned}$$

where $\mathrm{\Lambda }=\mathrm{\Sigma }_{a_{z}} {{\Pi }}_{z}^{a_{z}}$ is a blind measurement at time *t*. And when the above expression equals to zero (i.e., $N= 0$), the NCGD can be satisfied. In other words, when $N\ne 0$, NCGD can be violated (CGD can be satisfied), which means that the evolution does generate coherence, and turns such coherence into the populations measured at a later time. Now, we investigate the NCGD when the measurement is the projective measurement of Eq. (1). From Eqs. (1), (6) and (14), the *N* in Eq. (14) under the projective measurement can be obtained as

15 $$\begin{aligned} N=\left( \begin{array}{cc} \frac{1}{2} \alpha \sin ^2\tau \omega &{} 0 \\ 0 &{} -\frac{1}{2} \alpha \sin ^2\tau \omega \\ \end{array} \right) . \end{aligned}$$

From the above expression, we find that when one of the conditions is satisfied: (1) $\tau =\frac{\pi }{\omega }$; (2) $\alpha =0$, the NCGD will be satisfied. That is to say, in this situation, the evolution may not generate coherence, and cannot also turn such coherence into the populations measured at a later time. Next, we investigate the effects of coarsening measurement reference on the NCGD ($\Delta \ne 0$ and $\delta = 0$). From Eqs. (3), (6) and (14), we obtain *N* in Eq. (14) under the coarsening measurement in reference, which can be rewritten as $N_{\Delta }$ and denoted as

16 $$\begin{aligned} N_{\Delta } =\left( \begin{array}{cc} -\frac{1}{2} \left( \sqrt{1-e^{-\Delta ^2}}-1\right) \alpha \sin ^2\tau \omega &{} -\frac{1}{4} i e^{-\Delta ^2} \left[ e^{\Delta ^2} \left( \sqrt{1-e^{-\Delta ^2}}-1\right) +1\right] \alpha \sin 2 \tau \omega \\ \frac{1}{4} i e^{-\Delta ^2} \left[ e^{\Delta ^2} \left( \sqrt{1-e^{-\Delta ^2}}-1\right) +1\right] \alpha \sin 2 \tau \omega &{} \frac{1}{2} \left( \sqrt{1-e^{-\Delta ^2}}-1\right) \alpha \sin ^2\tau \omega \\ \end{array} \right) . \end{aligned}$$

It can be found from the above expression that if one of the conditions is satisfied: (1) $\tau =\frac{\pi }{\omega }$; (2) $\alpha =0$, the NCGD will not be violated. If the measurement in final resolution is coarsened (${{\Delta }}=0, \delta \ne 0$), $N_{\delta }$ can be obtained from Eqs. (2), (6) and (14) (see “Supplementary Information”). And then, we find that the NCGD will not be violated, if one of the conditions is satisfied: (1)$\tau =\frac{\pi }{\omega }$; (2) $\alpha =0$. We then list these non-violation conditions of the NCGD for projective and coarsening measurements in Table 1.

**Figure 2:**
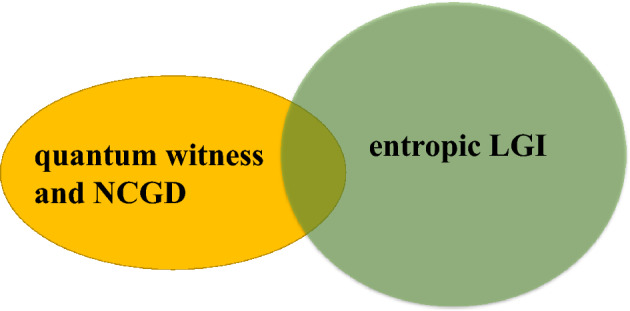
Schematic diagram for the relationship among the quantum witness, the entropic LGI and the NCGD, for the pure and dephasing qubits, in the case of the projective measurement, the coarsening measurement reference and the coarsening final measurement resolution. The shaded regions attributed to the quantum witness, the entropic LGI and the NCGD denote the non-violation conditions of the quantum witness, the entropic LGI and the NCGD for the pure and dephasing qubits, in the case of the projective measurement, the coarsening measurement reference and the coarsening final measurement resolution, respectively.

Now, we compare the quantum witness, the entropic LGI and the NCGD under projective and coarsening measurements for the pure qubit. It is clearly found from Table 1 that for the projective measurement, the non-violation conditions of the quantum witness and the NCGD are the same, while the non-violation conditions of the entropic LGI and the quantum witness do not contain each other. That is to say, the entropic LGI and the quantum witness complement each other, and their conjunction may be better for testing macrorealism. For the coarsening measurement reference and the coarsening final measurement resolution, we find that the relationship of the quantum witness, the entropic LGI and the NCGD is similar to that of the projective measurement (see Table 1). Then, we plot the relationship among the quantum witness, the entropic LGI and the NCGD in Fig. 2, for the pure qubit with the projective measurement, the coarsening measurement reference and the coarsening final measurement resolution.

## A qubit interacting with environment

As we are aware, quantum systems inevitably experience undesired interactions with their surrounding environment. Hence, in this section, we examine how the environment affects the non-violations of the quantum witness, the entropic LGI and the NCGD for the projective and the coarsening measurements. The time evolution of an open system differs from that of a closed system. In general, it cannot be described by a unitary time evolution. The system’s dynamics can be depicted by a suitable equation of motion for its density matrix, known as a quantum master equation. In this scenario, the system’s evolution is typically governed by the Lindblad form master equation, which can be expressed as follows:

17 $$\begin{aligned} \frac{d\rho }{d t}=-i[H, \rho ]+\sum _{k}\left[ 2 L_{k} \rho L_{k}^{\dagger }-L_{k}^{\dagger } L_{k} \rho -\rho L_{k}^{\dagger } L_{k}\right] , \end{aligned}$$

where $L_{k}$ represents the Lindblad operator, which characterizes the interaction between the system and its environment. Similar to the previous section, we introduce the Hamiltonian $H=\frac{1}{2}\omega \sigma _{x}$, which describes the coherent part of the dynamics.

### A dissipative qubit

Let’s examine the first scenario, and the Lindblad operator in Eq. (17) is defined as $L_k=\sqrt{\gamma }\sigma _{-}$, where $\gamma > 0$ represents the rate of spontaneous emission, and $\sigma _{-}=\vert 1\rangle \langle 0\vert$ represents the atomic lowering operator. For the quantum witness, from Eqs. (1), (5) and (17), all the quantum witnesses under the projective measurement, can be obtained. Similarly, we take $W_{q}=\mid P({{\Pi }}^{+}(t_2)) - \sum _{{\pm }} P({{\Pi }}^{\pm }(t_1),{{\Pi }}^{+}(t_2))\mid$ as an example, and $\mid P({{\Pi }}^{+}(t_2)) - \sum _{{\pm }} P({{\Pi }}^{\pm }(t_1),{{\Pi }}^{+}(t_2))\mid$ can be expressed as

18 $$ \begin{gathered}   \left| {P\left( {\Pi ^{ + } \left( {t_{2} } \right)} \right) - \sum\limits_{ \pm } P \left( {\Pi ^{ \pm } \left( {t_{1} } \right),\Pi ^{ + } \left( {t_{2} } \right)} \right)} \right| \hfill \\   \quad  = e^{{ - 4\gamma \tau }} \sin ^{2} \frac{{\omega \tau }}{2}\left[ {\left( {\alpha  + 1} \right)\left( {\cos \omega \tau  + 1} \right) + e^{{2\gamma \tau }} \cos \omega \tau  - e^{{4\gamma \tau }} \left( {2\cos \omega \tau  + 1} \right)} \right]. \hfill \\  \end{gathered}  $$

It is noted that the non-violation conditions of the quantum witness for the dissipative qubit are very complicated, thus, we suppose $\tau =\dfrac{\pi }{2\omega }$ to study the non-violations of it in the following. In that situation ($\tau =\dfrac{\pi }{2\omega }$), from Eq. (18), we find that if $\alpha =e^{\frac{2 \pi \gamma }{\omega }}-1$, the quantum witnesses will not be violated. Other quantum witnesses exhibit similar characteristics. Therefore, all of the quantum witnesses will not be violated, when $\alpha =e^{\frac{2 \pi \gamma }{\omega }}-1$. It is noted that $0\le \alpha \le 1$, i.e., $0\le e^{\frac{2 \pi \gamma }{\omega }}-1\le 1$, so $\gamma \in (0, \frac{\log 2}{2 \pi }\omega ]$. This non-violation condition is listed in Table 1. Next, we investigate the quantum witness under the coarsening measurement in reference (${{\Delta }}\ne 0, \delta =0$) and in final resolution (${{\Delta }}=0, \delta \ne 0$). Then, we obtain the quantum witness under the coarsening measurement reference and coarsening measurement final resolution (see “Supplementary Information”). Similarly, the non-violation conditions of the quantum witness are summarized in Table 1.

**Figure 3:**
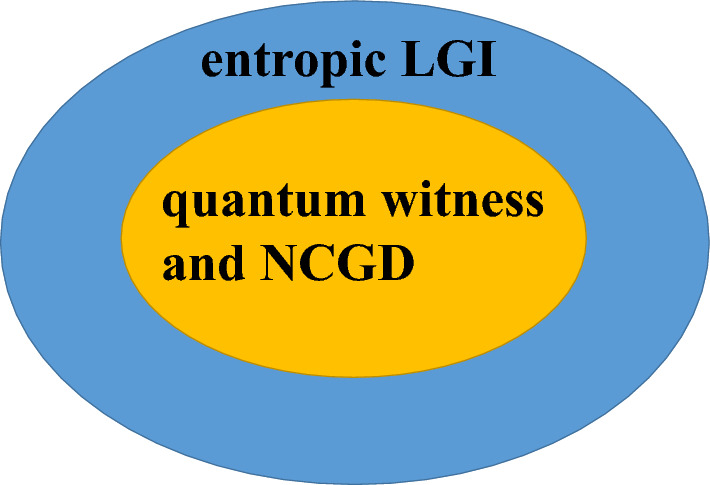
Schematic diagram for the relationship among the quantum witness, the entropic LGI and the NCGD, for the dissipative qubit with projective measurement. The shaded regions attributed to the quantum witness, the entropic LGI and the NCGD denote the non-violation conditions of the quantum witness, the entropic LGI and the NCGD for the dissipative qubit with projective measurement, respectively.

**Figure 4:**
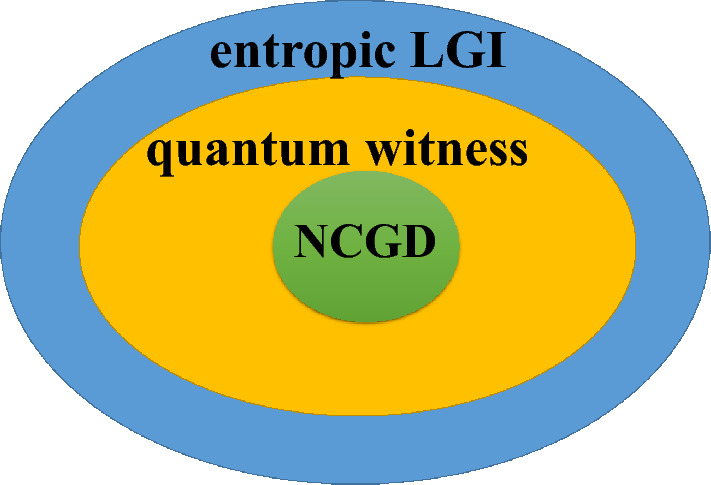
Schematic diagram for the relationship among the quantum witness, the entropic LGI and the NCGD, for the dissipative qubit, in the case of the coarsening measurement both in reference and in final resolution. The shaded regions attributed to the quantum witness, the entropic LGI and the NCGD denote the non-violation conditions of the quantum witness, the entropic LGI and the NCGD for the dissipative qubit with the coarsening measurement both in reference and in final resolution, respectively.

Next, we investigate the entropic LGI for the projective measurement. Additionally, we similarly assume that $\tau =\frac{\pi }{2\omega }$ to study non-violation conditions of it. Using Eqs. (1), (10) and (17), we calculate the entropic LGI for the projective measurement when $\tau =\frac{\pi }{2\omega }$ (see “Supplementary Information”). Because the non-violation conditions of the entropic LGI are numerous and complex, it is too cumbersome to list every term of non-violation conditions of the entropic LGI. For simplicity and comparison with the quantum witness, we only take three non-violation conditions as an example (which are listed in Table 1): (1) $\alpha =e^{\frac{2 \pi \gamma }{\omega }}-1$ ($\gamma \in (0, \frac{\log 2}{2 \pi }\omega ]$); (2) $\alpha =0$; (3) $\alpha =0.5$ and $0.00684\omega \le \gamma \le 0.50432\omega$. Then, we obtain the entropic LGI for the coarsening measurement in two scenarios: (1) when the measurement reference is coarsened (${{\Delta }}\ne 0, \delta =0$), and (2) when the final measurement resolution is coarsened (${{\Delta }}=0, \delta \ne 0$). Similarly, then, we summarize these non-violation conditions of the entropic LGI for the coarsening measurement reference and coarsening final measurement resolution in Table 1, respectively.

Then, we examine the NCGD in the context of projective measurement and coarsening measurement, as defined in Eqs. (1-3), respectively. For the projective measurement, by Eqs. (1), (14) and (17), we obtain *N* in Eq. (14) (see “Supplementary Information”), and in the following, we assume $\tau =\frac{\pi }{2\omega }$ to study the non-violation conditions of the NCGD. Then, we find that when $\alpha =e^{\frac{2 \pi \gamma }{\omega }}-1$ ($\gamma \in (0, \frac{\log 2}{2 \pi }\omega ]$), the NCGD will be satisfied. That is to say, under these circumstances, the system’s evolution does not generate coherence and cannot convert any existing coherence into measurable populations at a later time. Next, we obtain the NCGD under the coarsening measurement in reference (${{\Delta }}\ne 0, \delta =0$) and in final resolution (${{\Delta }}=0, \delta \ne 0$) (see “Supplementary Information”). Interestingly, we cannot find any circumstance to make the NCGD non-violated, whether the coarsening measurement occurs in the reference or the final resolution. Similarly, we summarize the non-violation conditions of the NCGD for both projective and coarsening measurements in Table 1.

Finally, let’s compare the quantum witness, the NCGD and the entropic LGI for the dissipative qubit (see Table 1), and then provide a summary of them. It can be clearly found from Table 1 that for the projective measurement, the entropic LGI can be satisfied for a wider parameter than the quantum witness and the NCGD. And the non-violation conditions of the quantum witness and the NCGD are the same. In other words, when the NCGD and the quantum witness are satisfied, the entropic LGI must be satisfied, and the relationship among the quantum witness, the entropic LGI and the NCGD can be seen in Fig. 3, for the projective measurement. For the coarsening measurement both in reference and in final resolution, we find from Table 1 that the entropic LGI can be satisfied for a wider parameter than the quantum witness, and the quantum witness can be satisfied for a wider parameter than the NCGD. And the relationship among the quantum witness, the entropic LGI and the NCGD is shown in Fig. 4, for the coarsening measurement reference and the coarsening measurement final resolution. In addition, it can be seen from Table 1 that for $\alpha =0.5$ and $\gamma =0.6\omega$, the entropic LGI under the coarsening measurement in reference can be satisfied when $0.20522\le \Delta <1$, and the entropic LGI under the coarsening measurement in final resolution can be satisfied when $0.01042\le \delta <0.5$. In other words, the entropic LGI for the coarsening measurement in final resolution can be violated for a narrower parameter than that of the coarsening measurement in reference. Therefore, in the case of the entropic LGI, the violation of the coarsening final measurement resolution for the dissipation qubit is more vulnerable than that of the coarsening measurement reference, which is similar to that of the pure qubit.

### A dephasing qubit

Let’s now turn to the second case, where the Lindblad operator is defined as $L_k=\sqrt{\gamma }\sigma _{z}$. Next, we obtain the non-violation conditions of the quantum witness, the entropic LGI and the NCGD for projective and coarsening measurements, and then, summarize these non-violation conditions in Table 1. It is noted that similar to the previous section, we do not list all the non-violation conditions of the entropic LGI. And the listed non-violation conditions of the entropic LGI in Table 1 are for the purpose of comparing with the quantum witness and the NCGD. Then, it can be seen from Table 1 that for the projective measurement and the coarsening measurement, the non-violation conditions of the quantum witness are the same as the NCGD, while the non-violation conditions of the quantum witness and the entropic LGI do not contain each other, i.e., the conjunction of the quantum witness and the entropic LGI may be better for testing macrorealism. The relationship of the quantum witness, the entropic LGI and the NCGD for projective and coarsening measurements, is similar to that of the pure qubit, which is shown in Fig. 2. In addition, it can be found in Table 1 that similar to that of the pure and dissipative qubits, in the case of the entropic LGI, the violation of the coarsening final measurement resolution for the dephasing qubit is more vulnerable than that of the coarsening measurement reference.

## Conclusions

In this paper, we detailed an analysis of the non-classical properties of three different criteria of quantumness, i.e., the quantum witness, the entropic LGI and the NCGD, under projective and coarsening measurements. The coarsening measurement can be divided into coarsening in measurement reference and coarsening in final measurement resolution. We consider a qubit in three scenarios: with coherent dynamics, dynamics with dissipation and dephasing. For the pure and dephasing qubits, we find that in the case of projective and coarsening measurements, the non-violation conditions of the quantum witness and the NCGD are the same; while in that situation, the non-violation conditions of the quantum witness and the entropic LGI do not contain each other, so a suitable conjunction of them may be better for testing macrorealism. Furthermore, for the pure qubit, the valve of the entropic LG function decreases as the coarsening degree of measurement reference increases, which is similar to that of the coarsening final measurement resolution. For the dissipative qubit with projective measurement, when the NCGD and the quantum witness are not violated, the entropic LGI must not be violated. For the dissipative qubit with coarsening measurement, the violation of the entropic LGI is more vulnerable than that of the quantum witness, and the violation of the quantum witness is more vulnerable than that of the NCGD. In addition, for the entropic LGI, we find that the robustness of the coarsening measurement reference is more than that of the coarsening measurement final resolution, in the systems with coherent dynamics and dynamics with dissipation and dephasing (see Table 1 and Table 2). In this paper, we take the closed and open systems to comparison of three different criteria of macrorealism, and expect that the results of this paper might be similar to some experimental situations. In addition, our investigation can help people understand the macrorealism from a different perspective. In the future, we will acquire a deeper understanding of it, and find more features for the logical connection among the quantum witness, the entropic LGI and the NCGD.

### Supplementary Information

Supplementary Information.

## Data Availability

All data generated or analysed during this study are included in this published article [and its supplementary information files].
