# Supplementary material for: Comparing the quantum witness, the entropic Leggett–Garg inequality and the NCGD
Source: Sci Rep. 2024 May 2;14:10139. doi: 10.1038/s41598-024-60742-y (PMC11066071; doi:10.1038/s41598-024-60742-y)
Supplement: Supplementary file 1 — Supplementary Information. [file 41598_2024_60742_MOESM1_ESM.zip › Supplementary/Supplementary.pdf]

Comparing the quantum witness, the entropic Leggett-Garg inequality and the NCGD

Xiangguan Tan   Yuxia Zhang *   Tianhui Qiu

A A pure qubit

A.1 NCGD

Using Eqs. (2), (6) and (14), we obtain N in Eq. (14) for the coarsening final measurement resolution, which can be rewritten as N_δ and expressed as

$$N_\delta = \begin{pmatrix} \frac{1}{2}\alpha\left[1 - 2\sqrt{-(\delta-1)\delta}\right]\sin^2\tau\omega & -\frac{1}{2}i\alpha\left[2(\delta-1)\delta + \sqrt{-(\delta-1)\delta}\right]\sin 2\tau\omega \\ \frac{1}{2}i\alpha\left[2(\delta-1)\delta + \sqrt{-(\delta-1)\delta}\right]\sin 2\tau\omega & \frac{1}{2}\alpha\left[2\sqrt{-(\delta-1)\delta} - 1\right]\sin^2\tau\omega \end{pmatrix}. \tag{A1}$$

It can be found that the NCGD will be satisfied, if one of the conditions is satisfied: (1) $\tau = \frac{\pi}{\omega}$; (2) $\alpha = 0$, which are listed in Table 1.

B A dissipative qubit

B.1 Quantum witness

Using Eqs. (2), (3), (5) and (17), the quantum witness for the coarsening measurement in reference and the coarsening measurement in final resolution, can be respectively expressed as

$$W_{q,\Delta} = \mid P_\Delta(\Pi^+(t_2)) - \sum_\pm P_\Delta(\Pi^\pm(t_1), \Pi^+(t_2)) \mid = e^{-\frac{\Delta^2}{2} - 4\gamma\tau}\sin^2\left(\frac{\tau\omega}{2}\right)$$

$$\times\left[\alpha + \cos\tau\omega\left(\alpha - (\alpha+1)\sqrt{1-e^{-\Delta^2}}e^{\gamma\tau} + \sqrt{1-e^{-\Delta^2}}e^{3\gamma\tau} + e^{2\gamma\tau} - 2e^{4\gamma\tau} + 1\right)\right.$$

*zhangyuxia0619@163.com

$$-(\alpha+1)\sqrt{1-e^{-\Delta^2}}e^{\gamma\tau}+\sqrt{1-e^{-\Delta^2}}e^{3\gamma\tau}-e^{4\gamma\tau}+1\Big], \tag{A2}$$

$$W_{q,\delta}=\mid P_\delta(\Pi^+(t_2))-\sum_{\pm}P_\delta(\Pi^\pm(t_1),\Pi^+(t_2))\mid=(2\delta-1)\left(-e^{-4\gamma\tau}\right)\sin^2\left(\frac{\tau\omega}{2}\right)$$
$$\times\Big[\alpha+\cos\tau\omega\left(\alpha-2(\alpha+1)\sqrt{-(\delta-1)\delta}e^{\gamma\tau}+2\sqrt{-(\delta-1)\delta}e^{3\gamma\tau}+e^{2\gamma\tau}-2e^{4\gamma\tau}+1\right)$$
$$-2(\alpha+1)\sqrt{-(\delta-1)\delta}e^{\gamma\tau}+2\sqrt{-(\delta-1)\delta}e^{3\gamma\tau}-e^{4\gamma\tau}+1\Big]. \tag{A3}$$

From Eqs. (A2) and (A3), we find the non-violation conditions of the quantum witness (with $\tau=\frac{\pi}{2\omega}$) for the coarsening measurement in reference and in final resolution, and then summarize them in Table 1.

B.2 Entropic LGI

From Eqs. (1), (10) and (17), the entropic LGI ($t=\frac{\pi}{2\omega}$) for the projective measurement can be obtained as

$$H_1=\frac{1}{2}\Big[(\alpha-1)\log\left(\frac{1-\alpha}{2}\right)-(\alpha-1)\log(1-\alpha)+(\alpha+1)\log(\alpha+1)-6\log 2 \tag{A4}$$
$$-(\alpha+1)e^{-\frac{2\pi\gamma}{\omega}}\left(\log[(\alpha+1)e^{-\frac{2\pi\gamma}{\omega}}]+(e^{\frac{2\pi\gamma}{\omega}}-1)\log[(\alpha+1)(1-e^{-\frac{2\pi\gamma}{\omega}})]-e^{\frac{2\pi\gamma}{\omega}}\log 2\right)\Big],$$

$$H_2=\frac{1}{2}\Big[(\alpha+1)e^{-\frac{2\pi\gamma}{\omega}}\left(\log[(\alpha+1)e^{-\frac{2\pi\gamma}{\omega}}]+(e^{\frac{2\pi\gamma}{\omega}}-1)\log[(\alpha+1)(1-e^{-\frac{2\pi\gamma}{\omega}})]\right)-(\alpha-1)\log(1-\alpha)\Big], \tag{A5}$$

$$H_3=\frac{1}{2}e^{-\frac{2\pi\gamma}{\omega}}\Big[-e^{\frac{2\pi\gamma}{\omega}}(\alpha+1)\log(\alpha+1)+(\alpha+1)(e^{\frac{2\pi\gamma}{\omega}}-1)\log[(\alpha+1)(1-e^{-\frac{2\pi\gamma}{\omega}})] \tag{A6}$$
$$-(-\alpha+2e^{\frac{2\pi\gamma}{\omega}}-1)\log[2-(\alpha+1)e^{-\frac{2\pi\gamma}{\omega}}]\Big].$$

It can be found from Eqs. (A4-A6) that the non-violation conditions of the entropic LGI for the projective measurement, and then we summarize them in Table 1.

B.3 NCGD

Using Eqs. (1), (14) and (17), N in Eq. (14) for the projective measurement can be written as

$$N = \begin{pmatrix} N_{11} & N_{12} \\ N_{21} & N_{22} \end{pmatrix}, \tag{A7}$$

where $N_{11} = \frac{1}{4}e^{-8\gamma\tau}\left[(-\alpha + e^{4\gamma\tau} - 1)\left((2e^{4\gamma\tau} - 1)\cos 2\tau\omega - 1\right) - 2e^{4\gamma\tau}(e^{4\gamma\tau} - 1)\cos\tau\omega\right]$, $N_{12} = N_{21} = 0$ and $N_{22} = \frac{1}{4}e^{-8\gamma\tau}\left[2e^{4\gamma\tau}(e^{4\gamma\tau} - 1)\cos\tau\omega - (-\alpha + e^{4\gamma\tau} - 1)\left((2e^{4\gamma\tau} - 1)\cos 2\tau\omega - 1\right)\right]$. For the coarsening measurement in reference ($\Delta \neq 0$ and $\delta = 0$), using Eqs. (3), (14) and (17), we obtain N in Eq. (14), which can be rewritten as N_Δ and described as

$$N_\Delta = \begin{pmatrix} N_{\Delta,11} & N_{\Delta,12} \\ N_{\Delta,21} & N_{\Delta,22} \end{pmatrix}, \tag{A8}$$

where

$$N_{\Delta,11} = \frac{1}{4}e^{-8\gamma\tau}\Big[-(-\alpha + e^{4\gamma\tau} - 1)\Big((\sqrt{1 - e^{-\Delta^2}}e^{2\gamma\tau} - 2e^{4\gamma\tau} + 1)\cos 2\tau\omega - e^{2\gamma\tau}\sqrt{1 - e^{-\Delta^2}} + 1\Big)$$
$$-2e^{4\gamma\tau}(e^{4\gamma\tau} - 1)\cos\tau\omega\Big],$$

$$N_{\Delta,12} = -\frac{1}{2}i\sqrt{1 - e^{-\Delta^2}}e^{-8\gamma\tau}\sin\tau\omega\left[(-\alpha + e^{4\gamma\tau} - 1)(\sqrt{1 - e^{-\Delta^2}}e^{2\gamma\tau} - 2e^{4\gamma\tau} + 1)\cos\tau\omega + e^{4\gamma\tau}(e^{4\gamma\tau} - 1)\right],$$

$$N_{\Delta,21} = \frac{1}{2}i\sqrt{1 - e^{-\Delta^2}}e^{-8\gamma\tau}\sin\tau\omega\left[(-\alpha + e^{4\gamma\tau} - 1)(\sqrt{1 - e^{-\Delta^2}}e^{2\gamma\tau} - 2e^{4\gamma\tau} + 1)\cos\tau\omega + e^{4\gamma\tau}(e^{4\gamma\tau} - 1)\right],$$

$$N_{\Delta,22} = \frac{1}{4}e^{-8\gamma\tau}\Big[(-\alpha + e^{4\gamma\tau} - 1)\Big((\sqrt{1 - e^{-\Delta^2}}e^{2\gamma\tau} - 2e^{4\gamma\tau} + 1)\cos 2\tau\omega - e^{2\gamma\tau}\sqrt{1 - e^{-\Delta^2}} + 1\Big)$$
$$+2e^{4\gamma\tau}(e^{4\gamma\tau} - 1)\cos\tau\omega\Big].$$

Then, from Eqs. (2), (14) and (17), we obtain N in Eq. (14) for the coarsening final measurement resolution, which can be rewritten as N_δ and given by

$$N_\delta = \begin{pmatrix} N_{\delta,11} & N_{\delta,12} \\ N_{\delta,21} & N_{\delta,22} \end{pmatrix}, \tag{A9}$$

where

$$N_{\delta,11} = \frac{1}{2}e^{-8\gamma\tau}\Big[e^{2\gamma\tau}(e^{4\gamma\tau} - 1 - \alpha)\Big(\sqrt{-(\delta - 1)\delta} + (e^{2\gamma\tau} - \sqrt{-(\delta - 1)\delta})\cos 2\tau\omega\Big) + (\alpha - e^{4\gamma\tau} + 1)\cos^2\tau\omega$$
$$+(e^{4\gamma\tau} - e^{8\gamma\tau})\cos\tau\omega\Big],$$

$$N_{\delta,12} = -i\sqrt{-(\delta - 1)\delta}e^{-8\gamma\tau}\sin\tau\omega\left[(-\alpha + e^{4\gamma\tau} - 1)(2\sqrt{-(\delta - 1)\delta}e^{2\gamma\tau} - 2e^{4\gamma\tau} + 1)\cos\tau\omega + e^{4\gamma\tau}(e^{4\gamma\tau} - 1)\right],$$

$$N_{\delta,21} = i\sqrt{-(\delta-1)\delta}e^{-8\gamma\tau}\sin\tau\omega\left[(-\alpha+e^{4\gamma\tau}-1)(2\sqrt{-(\delta-1)\delta}e^{2\gamma\tau}-2e^{4\gamma\tau}+1)\cos\tau\omega+e^{4\gamma\tau}(e^{4\gamma\tau}-1)\right],$$

$$N_{\delta,22} = \frac{1}{4}e^{-8\gamma\tau}\times\Big[(-\alpha+e^{4\gamma\tau}-1)\left((2\sqrt{-(\delta-1)\delta}e^{2\gamma\tau}-2e^{4\gamma\tau}+1)\cos 2\tau\omega-2\sqrt{-(\delta-1)\delta}e^{2\gamma\tau}+1\right)$$
$$+2e^{4\gamma\tau}(e^{4\gamma\tau}-1)\cos\tau\omega\Big].$$

From Eqs. (A7-A9), we find that the non-violation conditions of the NCGD for projective and coarsening measurements, and then, these non-violation conditions of it are summarized in Table 1.
